# Effects of a Home-Based Exercise Program Incorporating Mindfulness and Yoga on Balance and Mobility in People With Parkinson Disease: Protocol for a Randomized Controlled Trial

**DOI:** 10.2196/97781

**Published:** 2026-06-03

**Authors:** Jojo Yan Yan Kwok, Lily Man Lee Chan, Yuki Yuk Shum, Shirley Yin Yu Pang, Man Auyeung, Bastiaan R Bloem

**Affiliations:** 1 School of Nursing Li Ka Shing Faculty of Medicine University of Hong Kong Hong Kong China (Hong Kong); 2 Center on Behavioral Health Faculty of Social Sciences University of Hong Kong Hong Kong China (Hong Kong); 3 Department of Medicine Queen Mary Hospital Hong Kong China (Hong Kong); 4 Department of Medicine Pamela Youde Nethersole Eastern Hospital Hong Kong China (Hong Kong); 5 Donders Institute for Brain, Cognition and Behaviour Radboud University Medical Center The Netherlands, Gelderland The Netherlands

**Keywords:** mindfulness, yoga, Parkinson disease, balance, motor training, freezing of gait, fall

## Abstract

**Background:**

Approximately 80% of individuals with Parkinson disease (PD) experience impaired balance and mobility, contributing to freezing of gait, an increased risk of falls, and reduced health-related quality of life. Mind-body interventions, such as mindfulness and yoga, may address both motor and nonmotor symptoms by enhancing mind-body coordination and reducing stress, thereby offering a scalable approach to balance rehabilitation in PD.

**Objective:**

This study aims to evaluate the effects and acceptability of Mindfulness Yoga–Practice Awareness through Cognitive-Based Exercise (MY-PACE), a mobile health–delivered, home-based intervention, on balance, mobility, and psychological well-being in individuals with PD and balance impairment.

**Methods:**

This assessor-blinded, 2-arm, randomized waitlist-controlled trial enrolled 132 individuals with PD and balance impairment, randomized to either the MY-PACE group (n=66, 50%) or the control group receiving routine outpatient care (n=66, 50%). The MY-PACE group will complete a 12-week, Zoom-delivered program incorporating mindfulness, yoga, and mindful walking practices. The control group will receive the intervention after study completion. The primary outcome is functional balance, as measured by the Berg Balance Scale. Secondary outcomes include functional mobility, gait patterns, balance confidence, freezing of gait severity, motor symptoms, anxiety and depressive symptoms, cognitive function, mindfulness, health-related quality of life, and fall incidence. Assessments will occur at baseline, 3 months (T1), and 6 months (T2). Data will be analyzed using linear mixed-effects models under the intention-to-treat principle.

**Results:**

Ethics approval was obtained on September 18, 2019. A preliminary feasibility study with 10 participants was conducted between May and June 2020. The full randomized controlled trial was funded in January 2022 by the Early Career Scheme 2021-2022 of the Research Grants Council, Hong Kong. The trial was prospectively registered on December 10, 2021. Recruitment began in April 2022. As of manuscript submission, 132 participants have been enrolled. Data analysis has not yet commenced. The study protocol and statistical analysis plan follow the original prespecified design. The results are expected to be published in 2026.

**Conclusions:**

This trial will evaluate a telehealth-delivered mindful yoga intervention for improving balance and mobility in individuals with PD. By integrating cognitive-based mindful awareness and motor training, MY-PACE targets both motor and nonmotor contributors to balance impairment. If effective, it may represent a scalable intervention for individuals with PD and other populations with mobility limitations.

**Trial Registration:**

Chinese Clinical Trial Registry ChiCTR2100054145; https://tinyurl.com/4j8f8zvn

**International Registered Report Identifier (IRRID):**

DERR1-10.2196/97781

## Introduction

### Background

Parkinson disease (PD) is the second most common neurodegenerative disorder worldwide, affecting approximately 10 million people. With population aging and increased life expectancy, its prevalence is projected to double by 2040 [1]. As PD progresses, functional balance declines, and motor symptoms become increasingly resistant to dopaminergic therapy [2,3]. Balance impairment imposes a substantial burden on individuals with PD, contributing to falls, fall-related injuries and fractures, functional limitations, an increased risk of institutionalization, and mortality [4,5]. Given the high prevalence and serious consequences of balance dysfunction—and the limited effectiveness of pharmacological management—there is an urgent need for complementary, nonpharmacological interventions to improve functional balance in PD [6,7].

Balance impairment in PD arises from a complex and multifactorial pathophysiology involving both motor and nonmotor mechanisms [7]. Motor deficits include disturbed stepping and postural instability, while nonmotor factors, such as cognitive decline, attentional deficits, and anxiety, further compromise balance control [4,7]. Neurophysiological studies indicate that basal ganglia dysfunction disrupts activation of the supplementary motor area, leading to impaired motor preparation and abnormal movement execution [8,9]. In addition, reduced dual-tasking capacity and difficulty shifting attention among motor, cognitive, and limbic networks contribute to instability. Increasingly, researchers and stakeholders—including the UK National Institutes of Health—have emphasized the importance of addressing nonmotor contributors to functional balance [9,10]. Despite this recognition, relatively few rehabilitation approaches integrate both motor and nonmotor targets.

Beyond pharmacotherapy, clinical guidelines recommend professionally led, cueing-based exercise programs to improve balance and gait in PD [11]. For example, physiotherapist-led interventions using rhythmic auditory stimulation, such as metronome cueing, are widely implemented in Western countries, including Belgium and New Zealand [12,13]. These approaches aim to activate premotor cortical regions through external sensory input, thereby bypassing dysfunctional basal ganglia–supplementary motor area circuits to facilitate improved gait patterns. However, the benefits of continuous cueing often diminish over time [14], possibly because gait control reverts from a goal-directed strategy to automatic processing within the impaired basal ganglia network. Furthermore, externally driven cueing systems are difficult to sustain as ambulatory, real-world interventions that support patients across daily contexts. The implementation of professionally supervised programs can also be resource-intensive and costly, limiting accessibility for many individuals with PD. Although such interventions demonstrate short-term improvements, they fail to reach a substantial proportion of patients in need. Importantly, most cueing-based programs primarily target motor skill training, with comparatively limited attention to nonmotor contributors to balance. Modifiable factors, such as cognition and anxiety, play critical roles in postural control and should be addressed concurrently within comprehensive balance rehabilitation strategies.

Mindfulness, a cognitive-based therapeutic approach, cultivates present-moment awareness with openness and nonjudgmental acceptance [15]. A systematic review of neuroimaging studies indicates that mindfulness-based interventions are associated with increased activation in the insula and prefrontal cortex—regions implicated in attentional control and emotional regulation [16]. Emerging empirical evidence further supports the use of mindfulness-based interventions for stress reduction and motor symptom management in PD [17,18]. A 2019 randomized clinical trial (RCT) demonstrated that mindfulness yoga is safe and effective in reducing anxiety and improving motor symptoms in individuals with PD, supporting its role as a complementary therapeutic strategy [17]. A subsequent 3-arm RCT showed that mindfulness techniques, including meditation and yoga, significantly improved anxiety, motor symptoms, and health-related quality of life (HRQOL) compared with usual care, with additional reductions in inflammatory markers [18]. Meditation also demonstrated sustained benefits for depressive symptoms and motor outcomes at a 6-month follow-up. Qualitative findings from this trial indicated that mindful walking improved gait and boosted confidence in balance among participants with PD [19]. Collectively, these findings highlight the multidimensional biopsychosocial benefits of mindfulness-based interventions in PD. Mindfulness techniques may function as an internal cueing strategy by enhancing attentional control, cognitive flexibility, and emotional regulation—mechanisms directly relevant to postural control and balance. Therefore, integrating mindfulness practice with motor skill training may enhance the avenues through which individuals improve mind-body coordination and functional balance.

Building on this evidence, we developed a novel intervention—Mindfulness Yoga–Practice Awareness through Cognitive‑Based Exercise (MY‑PACE). Unlike conventional motor-focused rehabilitation programs, MY‑PACE concurrently targets motor performance, attentional regulation, and anxiety management. The program is designed to enhance participants’ awareness of body coordination, postural alignment, and movement control, while cultivating emotional stability to reduce anxiety-related interference with balance. MY‑PACE incorporates audio‑guided mindful walking to support structured self‑practice, facilitating the development of internal cueing strategies applicable to everyday mobility tasks. By integrating mindfulness training with movement-based exercise, MY‑PACE seeks to engage cognitive, emotional, and motor pathways simultaneously, thereby expanding the mechanisms through which individuals may improve mobility, cognitive control, and emotional regulation to support functional balance.

### Aims and Hypotheses

This study aims to do the following:

- Examine the effects of MY‑PACE, compared with usual care, on functional balance (primary outcome) and secondary outcomes, including functional mobility, balance confidence, freezing of gait (FOG) severity, motor symptoms, anxiety and depressive symptoms, cognitive function, mindfulness, HRQOL, and the incidence of falls over a 6‑month period in individuals with PD
- Explore participants’ experiences with MY‑PACE, including their practice of mindfulness yoga and factors influencing motivation, acceptability, and integration into daily life

It is hypothesized that participants with PD who receive the MY‑PACE intervention will demonstrate significantly greater improvements in functional balance at postintervention follow-up (T1) and at the 6‑month follow‑up (T2) compared with those receiving usual care. In addition, it is hypothesized that participants in the MY‑PACE group will show greater positive changes in secondary physical, psychological, and cognitive outcomes relative to the control group across the same time points.

The exploratory research question is as follows: What are participants’ experiences of using mindfulness yoga as a lifestyle intervention for balance management, particularly regarding perceived effects, perceived mechanisms of action (ie, how and why mindfulness may or may not work), and contextual factors influencing motivation, acceptability, and real‑life practice?

## Methods

### Study Design

**Figure 1:**
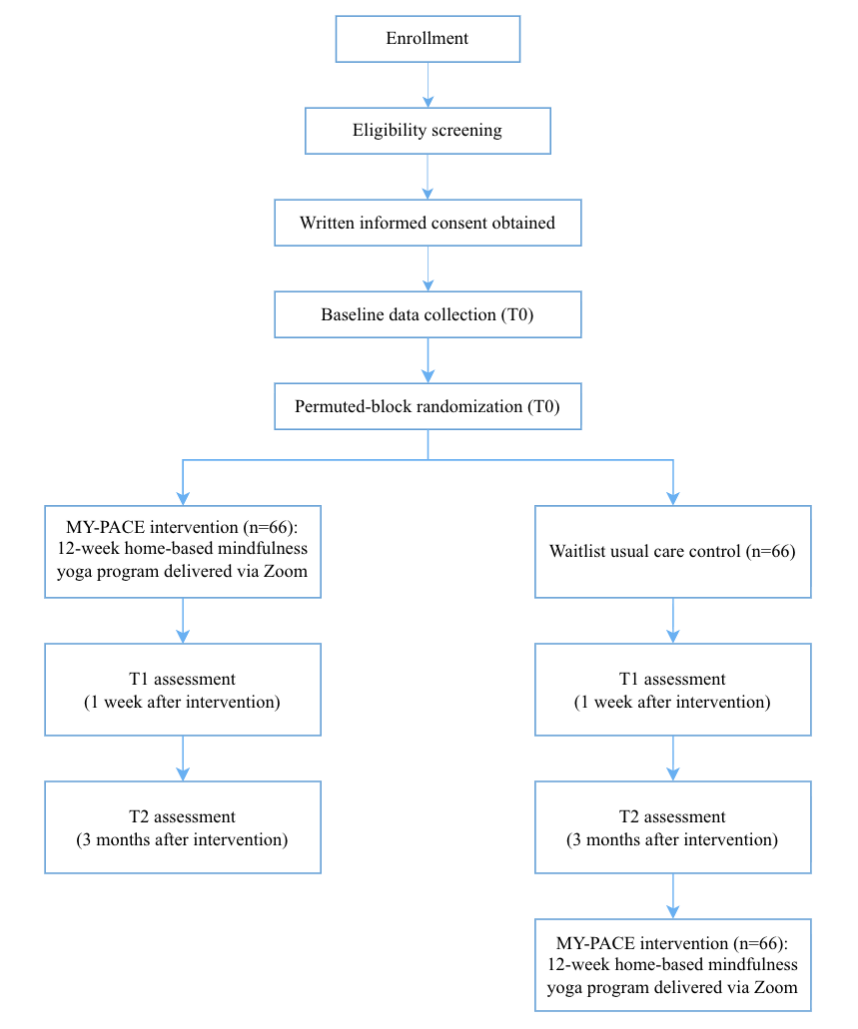
CONSORT (Consolidated Standards of Reporting Trials) flow diagram. MY-PACE: Mindfulness Yoga–Practice Awareness through Cognitive-Based Exercise.

This study is a multicenter, assessor-blinded, 2-arm RCT using a sequential explanatory mixed methods design to evaluate the effectiveness and acceptability of a 12-week home-based mindfulness yoga program (MY‑PACE) compared with usual care. The study flow is presented in Figure 1 (CONSORT [Consolidated Standards of Reporting Trials] flow diagram). Outcomes will be assessed at 3 time points over 6 months: baseline prior to randomization (T0), 1 week after intervention (T1), and 3 months after intervention (T2).

To complement the quantitative evaluation, individual semistructured qualitative interviews will be conducted at T1 with a purposive sample of 30 participants from the intervention group. Interviews will explore participants’ experiences with the MY‑PACE program, including perceived benefits, challenges, mechanisms of action, and views regarding the role of mindfulness yoga in managing PD-related symptoms. The sequential explanatory mixed methods design enables a comprehensive assessment of both intervention outcomes and implementation processes [20]. Quantitative analyses will determine the comparative effectiveness of MY‑PACE vs usual care, while qualitative findings will provide contextual insight into participant experiences, perceived mechanisms of change, and factors influencing engagement and adherence. Triangulation of quantitative and qualitative data will enhance understanding of how and why the intervention may influence balance, mobility, and related outcomes. The development-evaluation-implementation process of this study follows the Medical Research Council framework for complex interventions [21].

### Study Participants

Individuals were eligible for inclusion if they (1) had a diagnosis of idiopathic PD (Hoehn and Yahr stage I-III) and reported subjective balance or mobility difficulties [22] (this criterion was selected to capture early functional limitations that are relevant to patients’ perceived mobility challenges and daily functioning; objective measures of balance and mobility were collected at baseline to characterize impairment severity), (2) were aged ≥18 years, (3) were able to ambulate independently with or without an assistive device, (4) were able to provide written informed consent, and (5) had access to a smartphone or tablet device with an internet connection to participate in the online intervention. Individuals were excluded if they (1) were currently participating in another behavioral or pharmacological trial; (2) demonstrated significant cognitive impairment as indicated by the Hong Kong Montreal Cognitive Assessment 5‑minute protocol, using age- and education-adjusted normative cutoff scores [23,24]; or (3) had serious medical conditions other than PD, or any contraindication to mild‑to‑moderate physical exertion that would limit safe participation in the intervention (eg, severe visual or hearing impairment, unstable cardiovascular disease, or significant orthopedic conditions).

### Sample Size Calculation

Taking functional balance as the primary outcome, a meta‑analysis in individuals with PD reported a pooled mean difference of 3.64 (95% CI 2.06-5.22) points for resistance and balance training compared with control, corresponding to an estimated standardized effect size of Cohen *d*=0.66 [25], while a meta-analysis of cognitive-motor training in PD reported a standardized mean difference of 0.58 for balance outcomes compared with active or passive controls [26]. As this study will compare internally cued motor training with usual care, a conservative moderate effect size of 0.55 is assumed for the primary outcome. Using G*Power (version 3.1) for a 2‑arm parallel randomized controlled trial with a 2‑sided significance level of 0.05 and 80% power, 110 participants were required to detect an effect size of 0.55. Allowing for an anticipated attrition rate of 20%, a total sample size of 132 participants (66 per group) was recruited.

### Procedure

Participants were recruited from local outpatient clinics and community organizations serving individuals with PD, including the Hong Kong Parkinson’s Disease Foundation, the Hong Kong Parkinson’s Disease Association, and the Community Rehabilitation Network. Study information and promotional materials were disseminated through online platforms and organizational networks. Potential participants were contacted by telephone or WhatsApp (Meta Platforms Inc) and screened for eligibility. Eligible individuals received a detailed explanation of the study via videoconference. Written informed consent was obtained in person prior to data collection. Following consent, baseline demographic and clinical assessments were conducted. All outcome assessments were performed during the participants’ self-reported medication “on” state to minimize the influence of motor fluctuations. Assessments were administered by trained research personnel who were blinded to group allocation. Participants were then randomly assigned in a 1:1 ratio to either the MY-PACE group (mindfulness-based, internally cued motor training) or the usual care group using a computer-generated randomization sequence. The allocation sequence was prepared by a researcher not involved in recruitment, intervention delivery, or outcome assessment. Group allocation was communicated to participants by an independent staff member to ensure allocation concealment. All outcome assessors remained blinded to group assignment throughout the study period.

### Interventions

#### MY-PACE Program

Participants assigned to the MY‑PACE group receive 12 weekly, 90‑minute, live, supervised group sessions via Zoom (total virtual contact time: 18 hours), in addition to usual care. All participants are also instructed to practice the 20-minute audio-guided mindful walking exercise daily. Adherence is encouraged and documented through weekly phone calls or WhatsApp messages during the intervention period. Sessions are delivered to groups of approximately 20 participants by a certified yoga instructor with formal mindfulness qualifications. This protocol is adapted from a previously tested mindfulness yoga program implemented in a local population with PD [17,27] and comprises a combination of yoga movement, mindfulness meditation, and breathing techniques. This program emphasizes mobility and gait training, with a focus on mindful walking. Session content and structure are outlined in Multimedia Appendix 1.

#### Usual Care

The usual care group continues routine outpatient services and receives an educational leaflet on general fall prevention and FOG management. Participants in this group will be offered the MY-PACE program after completion of the 6‑month study period.

### Treatment Fidelity

Prior to intervention commencement, the instructor completed an 8‑hour training session covering protocol procedures and safety precautions, including a return demonstration to ensure adherence to the intervention manual. Treatment fidelity is evaluated through virtual on‑site observation conducted by an independent research assistant. Adherence to the protocol is assessed using a standardized observation checklist to determine whether all modules are delivered as specified.

### Outcome Measures

Assessments are conducted at baseline (T0), 1 week after intervention (T1), and 3 months after intervention (T2) for all participants.

#### Primary Outcome

Functional balance is measured using the Berg Balance Scale (BBS). The BBS comprises 14 items evaluating balance performance during common daily activities, each rated on a 5‑point scale (0-4), yielding a total score ranging from 0 to 56. Scores of 0 to 20 indicate severe balance impairment, 21 to 40 indicate moderate impairment, and 41 to 56 indicate good balance. The BBS demonstrates excellent interrater (intraclass correlation coefficient=0.98) and intrarater reliability (intraclass correlation coefficient=0.98) and has been validated in individuals with PD [28].

#### Secondary Outcomes

Functional mobility is assessed using a validated smartphone-based instrumental 3-meter Timed-Up-and-Go (TUG) test [17] via the US Food and Drug Administration–approved Mon4t Clinic (Montfort Brain Monitor Ltd) application [29]. A designated device is attached to the participant’s sternum using a flexible strap. Participants are instructed to sit on a chair with their hips positioned fully against the back of the seat. When the test starts, a countdown is heard, followed by an auditory and vibratory start cue. Participants are instructed to stand up, walk for 3 meters, turn around, walk back to the chair, and sit down. The total test time is recorded.

Gait patterns are assessed using a validated smartphone‑based 5‑meter instrumented TUG test via the Food and Drug Administration–approved Mon4t Clinic application. The device is positioned at the sternum as previously described. Participants complete 3 conditions: (1) a standard 5‑meter TUG test, in which they rise from a chair, walk 5 meters, turn, return, and sit down; (2) an obstacle 5‑meter TUG test, during which they walk 5 meters between 2 chairs spaced 24 inches apart before turning and returning to sit; and (3) a dual‑task 5‑meter TUG test. For the dual‑task condition, participants perform the 5‑meter TUG test while simultaneously completing a cognitive task involving serial subtraction performed continuously until the test is completed. Objective gait parameters, including step length, cadence, gait velocity, step-to-step variability, and postural sway, are extracted from the application.

Perceived balance confidence is measured in both medical “on” and “off” states using the Chinese version of the Activities‑Specific Balance Confidence Scale [30], a validated and reliable 16‑item instrument that evaluates individuals’ confidence in performing various indoor and outdoor activities. Each item is rated from 0% (no confidence) to 100% (complete confidence). Higher total scores indicate greater balance confidence.

Subjective experiences of FOG are assessed using the Chinese version of the FOG-Questionnaire [31]. The FOG‑Questionnaire consists of 6 items evaluating the severity and impact of freezing episodes on gait. Each item is rated on a 5‑point scale from 0 (absence of symptoms) to 4 (most severe), yielding a total score ranging from 0 to 24. Higher scores indicate more severe FOG.

PD-related motor symptoms are measured using the validated Movement Disorder Society–Unified Parkinson’s Disease Rating Scale (Chinese version) part III [32], which measures major PD motor symptoms, including tremor, rigidity, bradykinesia, gait, and postural instability. Higher scores indicate greater disease severity. The motor examination is conducted by qualified personnel with a Movement Disorder Society–Unified Parkinson’s Disease Rating Scale certificate.

Anxiety and depressive symptoms are measured using the validated Hospital Anxiety and Depression Scale (Chinese-Cantonese), a self-report questionnaire that consists of anxiety and depression subscales. Each subscale consists of 7 items, and each item is rated on a 4-point scale. A high score represents a high level of psychological distress. The Hospital Anxiety and Depression Scale has been suggested for use in populations with PD because somatic symptoms that may overlap with Parkinsonian manifestations are not assessed in this scale [33]. Additionally, the scale focuses on measuring the negative emotions related to anxiety and depression, which have been reported as the most prominent psychological factors in patients with PD.

Cognitive function and attention are measured using the Hong Kong Montreal Cognitive Assessment 5-min protocol, a validated tool for cognitive impairment in Chinese older adults that can be administered over the telephone. It consists of 4 subtests examining 5 cognitive domains, including attention, verbal learning and memory, executive functions or language, and orientation. Total scores range between 0 and 30. Patients with significant cognitive impairment are excluded using the cutoff scores based on age- and education-corrected normative data [23].

Mindfulness is measured using the validated 20-item Five-Facet Mindfulness Questionnaire (short-form; Chinese version) [34]. Using a 5-point Likert scale, 5 mindfulness domains are assessed, including observing, describing, acting with awareness, nonjudgment of inner experience, and nonreaction to inner experience.

HRQOL is assessed using the validated PD questionnaire-8 (Chinese version) [35,36]. With 8 items, the scale yields a summary index score capturing disease-specific HRQOL regarding mobility, activities of daily living, emotional well-being, social support, cognitions, communication, bodily discomfort, and stigma.

The incidence, reasons, and consequences of falls are assessed monthly. A fall is defined as “an unexpected event in which you come to rest on the ground, floor, or other lower level.” Participants are contacted monthly by a trained research assistant using a structured interview format. During each follow-up, participants are reminded of the standardized fall definition and asked the following standardized questions: (1) whether any falls occurred in the preceding month, (2) the number of falls, (3) the circumstances surrounding each fall, and (4) whether any injury or medical attention resulted. To reduce recall bias, participants are encouraged to record falls prospectively during the study period.

Sociodemographic data are collected using a self-report questionnaire, including age, gender, marital status, educational level, religiosity, and financial status. In addition, clinical data, including time since onset, history of comorbidities (eg, hypertension and diabetes mellitus), history of psychiatric disturbances, treatment and medication records, and history of rehabilitation service use, are documented and considered control variables in the data analysis.

### Ethical Considerations

This study complies with the ethical principles set forth in the Declaration of Helsinki. Approval was granted by the institutional review board of the University of Hong Kong–Hospital Authority Hong Kong West Cluster (UW 19-535) on September 18, 2019. The study objectives, voluntary participation, and participants’ rights—including the right to decline participation and withdraw at any time without any consequence—were communicated clearly both verbally and through a detailed information sheet. Participants are encouraged to ask questions and seek clarification about the study to ensure they fully understand their rights. Each participant is assigned a participant code to ensure anonymity, and no identifying information is included in the questionnaires. All collected data are securely stored and are accessible only to members of the research team. To enhance the protection of participants’ confidentiality, no identifiable information is included in the data files. All data will be destroyed 7 years after the study completion. Modest financial incentives (US $6.4, $6.4, and $12.8 at baseline, T1, and T2, respectively) will be provided to participants who completed the assessment interviews.

### Safety

Safety is of particular importance in individuals with PD, especially given the increased risk of falls. Appropriate measures are implemented to ensure participant safety during both exercise sessions and assessment time points. Participants identified as being at risk of falls are instructed to perform the live‑streamed MY‑PACE sessions in a safe environment, positioned next to a wall for support, with a stable chair placed nearby. Caregivers are encouraged to remain present during exercise sessions to provide additional supervision and assistance if necessary. Participants are instructed to immediately report any adverse events or discomfort related to the intervention to the research team. All adverse events are documented systematically, monitored throughout the study period, and followed by appropriate actions to ensure participant safety.

### Data Analysis

Data analysis will be performed using SPSS (version 28.0.1; IBM Corp) software following the intention-to-treat approach. Descriptive statistics will be used to summarize the sociodemographic characteristics and clinical background of all participants. The homogeneity of baseline characteristics between the 2 groups will be tested by the Pearson chi-squared test or Fisher exact test, as appropriate. Linear mixed-effect models will be used to assess the intervention effects on the primary and secondary outcomes, with time (as a continuous variable), group, and the group-by-time interaction included as independent variables. Linear contrasts will be used to obtain between-group differences. The normality of the residuals and random effects will be assessed using normal probability plots. Missing values will not be replaced because mixed-effect models can accommodate participants with at least 1 outcome measurement [37]. A 5% level of significance will be adopted, with all significance tests being 2-sided.

For the qualitative data, all audio-taped interviews will be transcribed verbatim and managed by NVivo (version 12.0; Lumivero LLC). Two experienced qualitative researchers will carefully and inductively review the transcripts. A 5-step thematic analysis will be adopted [38]. The coded units will be sorted into categories and subcategories and analyzed to identify recurrent themes and patterns. This qualitative data will enhance the interpretation of the quantitative findings and help identify the strengths and weaknesses of the intervention being tested. An audit trail will be maintained to ensure data credibility. The coded data will be compared to identify discrepancies, and any disagreements will be resolved through discussion.

## Results

Ethics approval was obtained on September 18, 2019. A small feasibility study (n=10) was conducted between May and June 2020 to inform the grant application for a full randomized controlled trial [39]. The study was subsequently funded by the Early Career Scheme 2021-2022 of the Research Grants Council, Hong Kong, with a funding period from January 1, 2022, to December 31, 2025. Prospective clinical trial registration was completed on December 10, 2021, prior to the start of recruitment in April 2022. As of manuscript submission, 132 participants have been enrolled. No outcome data analyses have been conducted to date. The submitted protocol reflects the original prespecified study design and statistical analysis plan. The trial results are expected to be published in 2026.

## Discussion

### Overview of the Study

PD is a progressive and irreversible neurodegenerative disorder affecting both motor and psycho‑cognitive function. Among its most disabling manifestations, functional balance impairment substantially contributes to falls, injuries, loss of independence, institutionalization, and mortality [4]. Despite its clinical importance, postural instability remains inadequately managed, particularly given the limited responsiveness of balance dysfunction to dopaminergic therapy. Therefore, there is a critical need for complementary, nonpharmacological strategies for functional balance rehabilitation.

Increasing evidence suggests that balance impairment in PD reflects disrupted integration among motor, attentional, and emotional processes rather than a purely motor deficit [4,8,9]. However, current rehabilitation guidelines continue to prioritize professionally led, externally cued exercise programs to improve gait and balance [11]. Rhythmic auditory stimulation and other cueing strategies are designed to facilitate movement by recruiting premotor cortical regions to compensate for basal ganglia dysfunction [12,13]. Although these approaches produce short-term improvements, their effects often diminish once external cues are withdrawn [14], raising concerns about long-term sustainability. Moreover, therapist‑dependent models are resource‑intensive and challenging to implement as continuous, real‑world support. Most externally cued interventions primarily target motor performance, with comparatively limited emphasis on modifiable nonmotor contributors, such as awareness training and anxiety management—factors that may underlie inconsistent translation of gains achieved in a structured setting into durable functional improvements.

The MY‑PACE program was designed to close critical gaps in PD rehabilitation by uniting mindfulness‑based attentional training with balance‑specific motor exercise within a whole‑person, person‑centered framework. Mindfulness practice cultivates present‑moment awareness and a nonjudgmental attentional stance, processes associated with enhanced attentional control and emotional regulation [16]. These processes may strengthen top‑down regulation of movement and emotional responses, allowing mindfulness techniques to function as internal cueing strategies that support intentional movement control, coordination, and postural alignment. In parallel, structured yoga‑based practice progressively trains balance, mobility, and mind-body coordination. Through this integrated approach, MY‑PACE engages motor, cognitive, and emotional domains that are foundational to functional performance and lived well‑being in PD. This trial evaluates a home‑based, interactive online delivery of MY‑PACE using a rigorous randomized controlled design. Effectiveness is tested with the BBS (primary outcome) and complemented by measures of mobility, psycho‑cognitive well‑being, and HRQOL. The repeated‑measures design, combined with participants’ experiential feedback, enables a comprehensive whole‑person evaluation of both impact and acceptability.

This study also tests a pragmatic tele‑rehabilitation model built for real‑world adoption. Traditional center‑based programs are constrained by transportation, cost, caregiver availability, and mobility limitations. Live‑streamed group sessions preserve structured guidance, peer connection, and real‑time feedback while markedly expanding reach. Daily self‑practice (audio‑guided mindful walking) embeds internally cued strategies into everyday contexts, increasing ecological validity and supporting sustained self‑management. By simultaneously targeting motor performance and key nonmotor drivers—especially attentional regulation and anxiety—MY‑PACE addresses the multidimensional nature of balance and mobility dysfunction in PD. If effective, this mindfulness‑based lifestyle program may be readily implementable within community rehabilitation services in Hong Kong and scalable across diverse health care systems. Findings from this trial can shape complementary rehabilitation pathways, advance priorities in fall prevention and mobility preservation, and inform chronic disease adaptation strategies. More broadly, this work contributes to accessible, community‑based, compassion‑focused models of PD care that align clinical benefits with patient values and everyday life.

### Limitations

Several limitations should be acknowledged. Despite using diverse recruitment strategies across clinical and community settings, participants who enrolled may have been more socially active, motivated, or technologically competent, introducing potential selection bias and limiting generalizability to individuals who are less engaged or more functionally impaired. Blinding of participants is not feasible because of the behavioral nature of the intervention, which introduces potential risks of performance and expectancy bias. To mitigate these effects, participants will not be informed of specific study hypotheses or detailed intervention content, the informed consent process will adhere to the principle of equipoise, and participants will be reminded not to disclose group allocation to outcome assessors to preserve assessor blinding. Importantly, the primary outcome (BBS) and several secondary mobility outcomes (eg, instrumented TUG tests and gait parameters) are performance-based and evaluated by blinded assessors or objectively quantified using instrumented measures, which may reduce susceptibility to expectation effects. Nevertheless, treatment expectancy and perceived intervention credibility are not formally assessed, limiting the ability to directly quantify potential expectancy influences. Incorporating validated measures of treatment expectancy and credibility in future studies may further strengthen the causal interpretation of between-group differences. Falls will be collected through monthly self-report and may therefore be subject to recall bias, despite the use of structured follow-up procedures and a standardized fall definition. Attrition is another methodological concern, as completion of 12 intervention sessions and 3 assessment time points may impose a participation burden. To enhance retention, flexible scheduling will be offered where feasible, control group participants will be offered the tested yoga intervention after completing follow-up assessments and modest financial incentives.

### Conclusions

This study tackles the widespread and disabling problem of balance impairment in PD by testing an internally cued, mindfulness‑integrated motor training paradigm delivered via telehealth. Leveraging growing evidence for mindfulness in PD, this RCT rigorously evaluates effects on balance, mobility, and psycho‑cognitive outcomes using validated objective and self‑report measures, complemented by participants’ experiential insights. By assessing both effectiveness and acceptability, this trial will clarify how mindfulness‑based motor training may enhance balance performance and the underlying regulatory processes that sustain it. Equally important, the study evaluates feasibility and real‑world reach through a digitally delivered, home‑based tele‑rehabilitation model that preserves structured guidance, peer connection, and real‑time feedback while reducing access barriers. Grounded in a whole‑person ethos that centers dignity, autonomy, and lived well‑being, the intervention integrates motor, cognitive, and emotional health aims within everyday life. Collectively, the findings will inform the clinical value, scalability, and health system integration of mind-body tele‑rehabilitation as a community‑based strategy for PD. The resulting evidence base can accelerate adoption of accessible, scalable, and compassion‑focused mindfulness‑based lifestyle interventions that help people with PD maintain stability, independence, quality of life, and dignity.

## Appendix

Multimedia Appendix 1 Themes and outlines of the 12-week Mindfulness Yoga–Practice Awareness through Cognitive-Based Exercise (MY-PACE) program.

## Abbreviations

BBS: Berg Balance Scale
CONSORT: Consolidated Standards of Reporting Trials
FOG: freezing of gait
HRQOL: health-related quality of life
MY-PACE: Mindfulness Yoga–Practice Awareness through Cognitive-Based Exercise
PD: Parkinson disease
RCT: randomized clinical trial
TUG: Timed-Up-and-Go Test
