# Supplementary material for: Effects of a Home-Based Exercise Program Incorporating Mindfulness and Yoga on Balance and Mobility in People With Parkinson Disease: Protocol for a Randomized Controlled Trial
Source: JMIR Res Protoc. 2026 Jun 3;15:e97781. doi: 10.2196/97781 (PMC13276467; doi:10.2196/97781)
Supplement: Multimedia Appendix 1 [file resprot_v15i1e97781_app1.docx]

**Multimedia Appendix 1.** Themes and outlines of the 12-week Mindfulness Yoga–Practice Awareness through Cognitive-Based Exercise (MY-PACE) program.

| **Program components** | **Week 1** | **Week 2** | **Week 3** | **Week 4** | **Week 5** | **Week 6** |  |
| --- | --- | --- | --- | --- | --- | --- | --- |
| Theme | - Introduction to the course: What is mindfulness? | - Nonjudging | - Beginner’s mind | - Patience | - Trust | - Non-Striving |  |
| Outline | - Meditation - Mindful breathing - Warm-up (seated) - Mountain pose - side-bend pose - forward-bend pose - neck stretches - twisting - shoulder stretch | - Meditation - Mindful breathing - Warm-up (as in week 1) | - Meditation - Mindful breathing - Warm-up (as in week 1) | - Meditation - Mindful breathing - Warm-up (as in week 1) | - Meditation - Mindful breathing - Warm-up (as in week 1) | - Meditation - Mindful breathing - Warm-up (as in week 1) |  |
| Mind-body movement practice | - Yoga sequence: - Cat-Cow pose - Modified sun salutation (on chair) - Upward Salute pose - forward-bend pose - Warrior I + waist twists - Warrior II - Mindful walking: lift, push, put down, and weight shift - Cool-down stretching (supine) - knee to chest - waist stretches - Savasana | - Yoga sequence - Cat-Cow pose - Standing side-bend pose - waist twists - Warrior III with support - Modified sun salutation (standing) - Upward Salute pose - half forward-bend pose - Warrior I - Warrior II - Mountain pose - Mindful walking - Cool-down stretching (seated) - waist twists - knee to chest - ankle stretches - Savasana | - Yoga sequence - Cat-Cow pose - side-bend pose - waist twists - wide-Legged Forward-Bend pose - Modified sun salutation (as in week 2) - Mindful walking - Cool-down stretching (seated or supine^a^) - Savasana | - Yoga sequence - Extended Cat-Cow pose - Child’s pose - waist twists - Warrior III with support - Modified sun salutation - Upward Salute pose - half forward-bend pose - Warrior I - Warrior II - Extended Side Angle Pose - Mountain pose - Mindful walking - Cool-down stretching (seated or supine^a^) - Savasana | - Yoga sequence - Extended Cat-Cow pose - Child’s pose - modified side-bend pose - wide-Legged Forward-Bend pose + twisting - Modified sun salutation (as in week 5) - Mindful walking - Cool-down stretching (seated or supine^a^) - Savasana | - Yoga sequence - Extended Cat-Cow pose - Child’s pose - Chair pose - Warrior III with support - Modified sun salutation - Upward Salute pose - half forward-bend pose - Warrior I - Warrior II - Extended Side Angle Pose - Reverse Warrior pose - Mountain pose - Mindful walking - Cool-down stretching (seated or supine^a^) - Savasana |  |
| **Program components** | **Week 7** | **Week 8** | **Week 9** | **Week 10** | **Week 11** | **Week 12** | |
| Theme | - Acceptance | - Letting Go | - Gratitude | - Summary of the previous 8 attitudes and application in daily life | - Summary of the previous 8 attitudes and application in daily life | - Summary of the previous 8 attitudes and application in daily life | |
| Outline | - Meditation - Mindful breathing - Warm-up (as in week 1) | - Meditation - Mindful breathing - Warm-up (as in week 1) | - Meditation - Mindful breathing - Warm-up (as in week 1) | - Meditation - Mindful breathing - Warm-up (as in week 1) | - Meditation - Mindful breathing - Warm-up (as in week 1) | - Meditation - Mindful breathing - Warm-up (as in week 1) | |
| Mind-body movement practice | - Yoga sequence - Extended Cat-Cow pose - Mountain pose - side-bend variation - Warrior III with support - Modified sun salutation (as in week 6) - Mindful walking - Cool-down stretching (seated or supine^a^) - Savasana | - Yoga sequence - Extended Cat-Cow pose - Child’s pose - Downward-Facing Dog pose - side-bend variation - wide-legged forward-bend pose + twisting - Modified sun salutation (as in week 6) - Mindful walking - Cool-down stretching (seated or supine^a^) - Savasana | - Yoga sequence - Extended Cat-Cow pose - Child’s pose - side-bend variation - Chair pose - Tree pose - Modified sun salutation (as in week 6) - Mindful walking - Cool-down stretching (seated or supine^a^) - Savasana | - Yoga sequence^b^ - Extended Cat-Cow pose - Child’s pose - Downward-Facing Dog pose - side-bend variation - Chair pose - wide-legged forward-bend pose + twisting - Warrior III with support - Tree pose - Modified sun salutation (as in week 6) - Mindful walking - Cool-down stretching (seated or supine^a^) - Savasana | - Yoga sequence^b^ - Extended Cat-Cow pose - Child’s pose - Downward-Facing Dog pose - side-bend variation - Chair pose - wide-legged forward-bend pose + twisting - Warrior III with support - Tree pose - Modified sun salutation (as in week 6) - Mindful walking - Cool-down stretching (seated or supine^a^) - Savasana | - Yoga sequence^b^ - Extended Cat-Cow pose - Child’s pose - Downward-Facing Dog pose - Side-bend variation - Chair pose - wide-legged forward-bend pose + twisting - Warrior III with support - Tree pose - Modified sun salutation (as in week 6) - Mindful walking - Cool-down stretching (seated or supine^a^) - Savasana | |

^a^Participants could choose either a supine or seated position according to their preference.
^b^Instructor would select appropriate yoga sequence according to participants’ condition.
